# Supplementary material for: The interactome of intact mitochondria by cross-linking mass spectrometry provides evidence for coexisting respiratory supercomplexes
Source: Mol Cell Proteomics. 2017 Dec 8;17(2):216–32. doi: 10.1074/mcp.RA117.000470 (PMC5795388; doi:10.1074/mcp.RA117.000470)
Supplement: Supplemental Data [file supp_RA117.000470_133922_0_supp_23978_fzffwf.zip › spectra_annotation/mito_spectra_annotation/395-1-11-1-9-1.pdf]

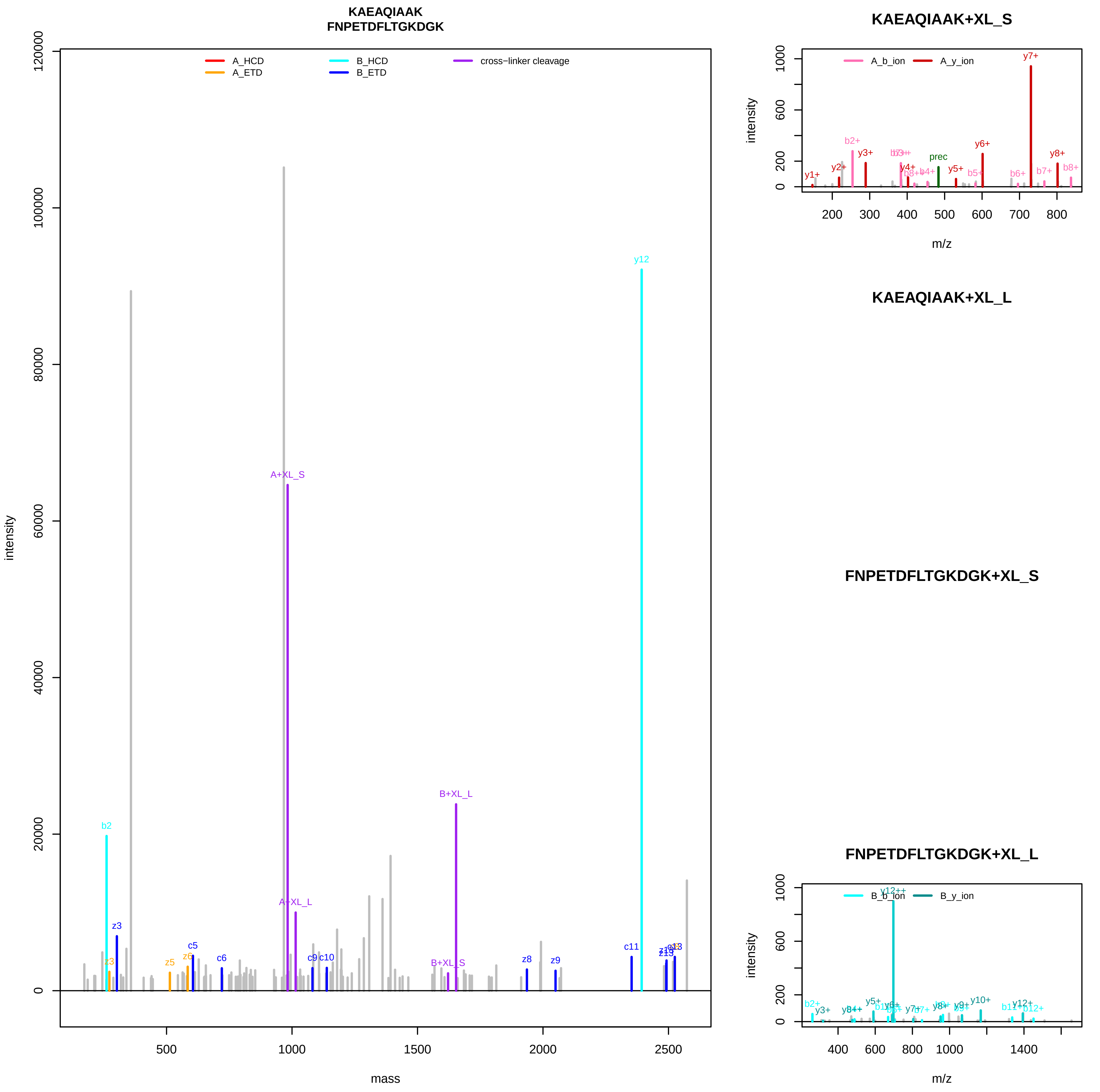
KAEAQIAAK
FNPETDFLTGKDGK
A_HCD
A_ETD
B_HCD
B_ETD
cross−linker cleavage
intensity
mass
y12
A+XL_S
B+XL_L
A+XL_L
B+XL_S
b2
z3
c5
c6
c9
c10
z8
z9
c11
z5
z6
KAEAQIAAK+XL_S
A_b_ion
A_y_ion
y7+
y6+
y3+
y2+
y1+
b2+
prec
y5+
y8+
b8+
b7+
b6+
b5+
b4+
y4+
m/z
KAEAQIAAK+XL_L
FNPETDFLTGKDGK+XL_S
FNPETDFLTGKDGK+XL_L
B_b_ion
B_y_ion
y12++
y10+
y9+
y8+
y7+
y6+
y5+
y3+
b2+
b12+
y12+
